# Supplementary material for: High-Strength, Degradable and Recyclable Epoxy Resin Based on Imine Bonds for Its Carbon-Fiber-Reinforced Composites
Source: Materials (Basel). 2023 Feb 15;16(4):1604. doi: 10.3390/ma16041604 (PMC9963643; doi:10.3390/ma16041604)
Supplement: Supplementary file 1 [file materials-16-01604-s001.zip › materials-2058581-supplementary.pdf]

materials

MDPI

Supporting Information

High strength, degradable and recyclable epoxy resin based on imine bonds for its carbon fiber reinforced composites

Yue Jiang[1], Shuai Wang[1], Weifu Dong[1], Tatsuo Kaneko[1,2], Mingqing Chen[1*] and Dongjian Shi[1*]

[1] Key Laboratory of Synthetic and Biological Colloids, Ministry of Education, School of Chemical and Material Engineering, Jiangnan University, Wuxi 214122, China

[2] Graduate School of Advanced Science and Technology, Japan Advanced Institute of Science and Technology, Ishikawa, 923-1292, Japan

* Correspondence: mqchen@jiangnan.edu.cn (M.C.); djshi@jiangnan.edu.cn (D.S.); Tel.: +86-510-85917019

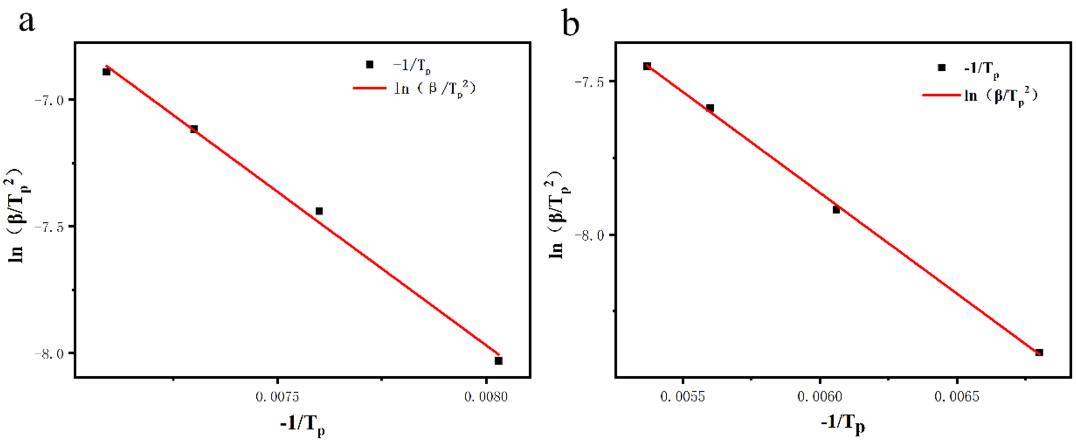

Figure S1. The curves fitted the Kissinger formula of (a) VAN-AC-EP/DDM and (b) DGEBA/DDM.

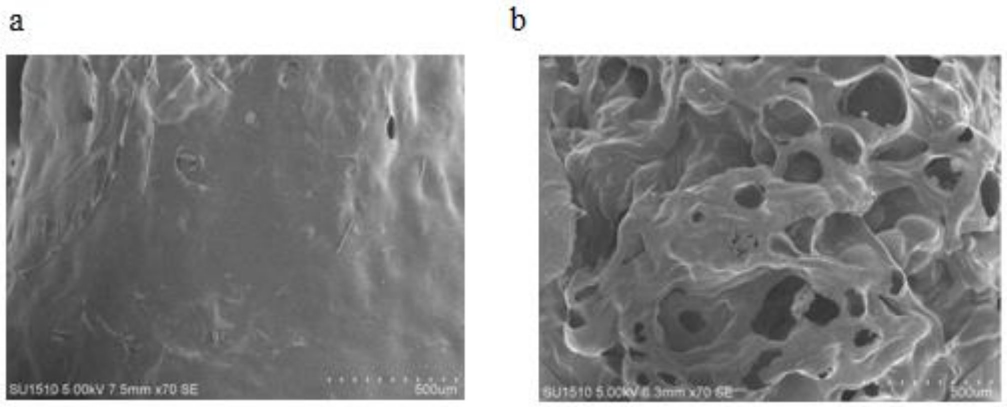

Figure S2. SEM images of carbon residue (a) VAN-AC-EP/DDM and (b) DGEBA/DDM .

Table S1. Tensile datas of resin and CFRP.

Sample	Tensile strength (MPa)	Young's Modulus (MPa)	Elongation at break (%)
VAN-AC-EP	44±2.2	2685±107	2.4±0.09
DGEBA	38±1.8	1966±117	2.9±0.15
Recycled VAN-AC-EP	29±1.3	2979±148	1.1±0.06
CFRP	122±7.3	6598±290	6.1±0.26
Recycled CFRP	82±4.1	9871±592	1.9±0.07

Table S2. Mechanical properties of thermosetting resin.

Sample	T_g (°C)	E_r (MPa)	T_r (K)	V_e (mol/m3)	Stiffness (kN/m)
VAN-AC-EP	146	6.1	459	568	588
DGEBA	202	26.6	519	2078	303

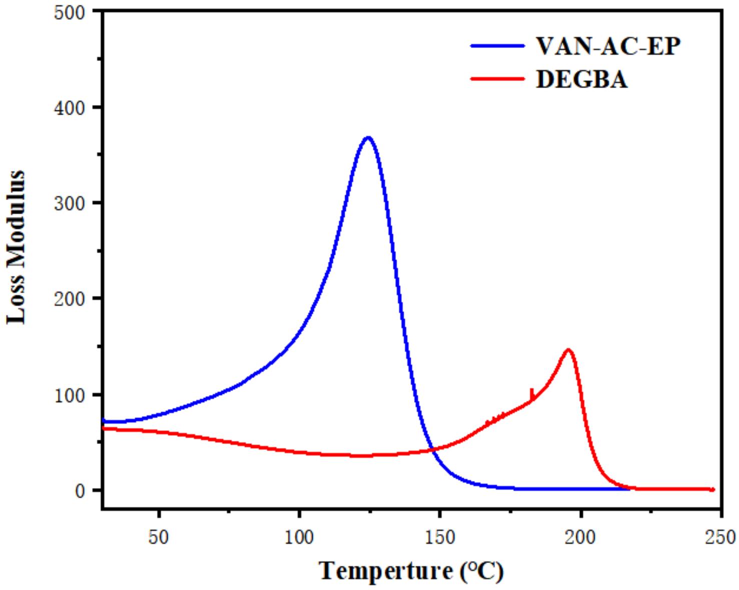

Figure S3. Loss modulus curves of VAN-AC-EP and DEGBA.

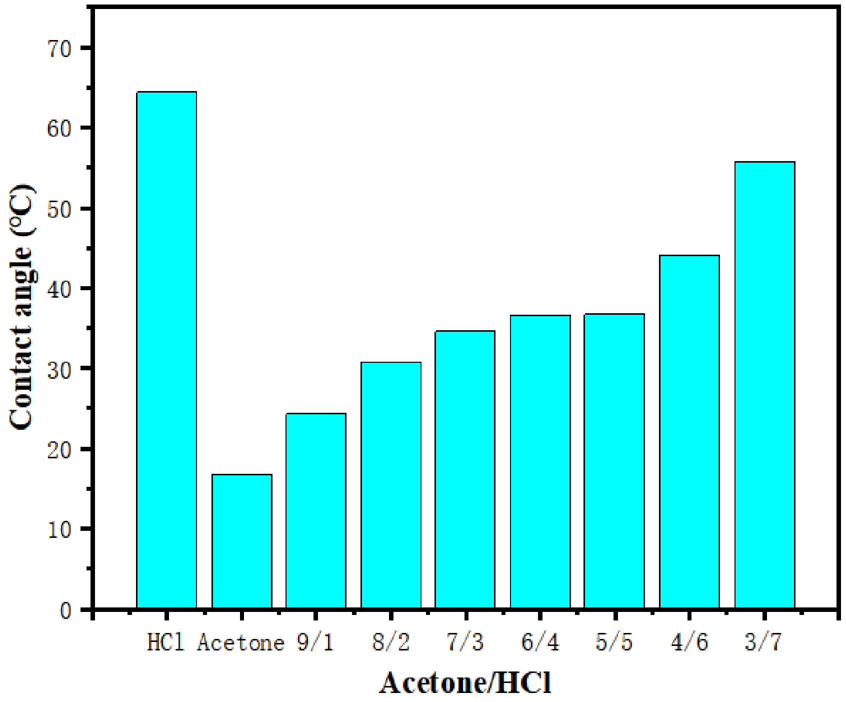

Figure S4. Contact angles of VAN-AC-EP/DDM in different ratios of acetone hydrochloric acid solution.

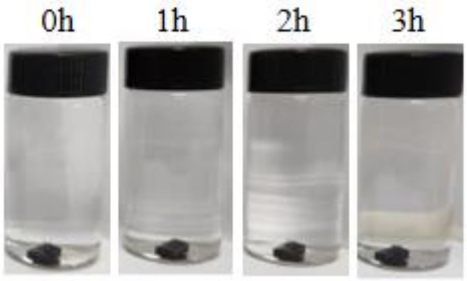

Figure S5. VAN- AC-EP/DDM was immersed in acetic acid solution.
